# Beneficial Effects of Human Mesenchymal Stromal Cells on Porcine Hepatocyte Viability and Albumin Secretion

**DOI:** 10.1155/2018/1078547

**Published:** 2018-01-11

**Authors:** Elisa Montanari, Joel Pimenta, Luca Szabó, François Noverraz, Solène Passemard, Raphael P. H. Meier, Jeremy Meyer, Jonathan Sidibe, Aurelien Thomas, Henk-Jan Schuurman, Sandrine Gerber-Lemaire, Carmen Gonelle-Gispert, Leo H. Buhler

**Affiliations:** ^1^Department of Surgery, Geneva University Hospitals and Medical Faculty, 1211 Geneva, Switzerland; ^2^Institute of Chemical Sciences and Engineering, Ecole Polytechnique Fédérale de Lausanne, 1015 Lausanne, Switzerland; ^3^University Centre of Legal Medicine, Universities of Lausanne and Geneva, Lausanne, Switzerland

## Abstract

Porcine hepatocytes transplanted during acute liver failure might support metabolic functions until the diseased liver recovers its function. Here, we isolated high numbers of viable pig hepatocytes and evaluated hepatocyte functionality after encapsulation. We further investigated whether coculture and coencapsulation of hepatocytes with human multipotent mesenchymal stromal cells (MSC) are beneficial on hepatocyte function. Livers from 10 kg pigs (*n* = 9) were harvested, and hepatocytes were isolated from liver suspensions for microencapsulation using alginate and poly(ethylene-glycol)- (PEG-) grafted alginate hydrogels, either alone or in combination with MSC. Viability, albumin secretion, and diazepam catabolism of hepatocytes were measured for one week. 9.2 ± 3.6 × 10^9^ hepatocytes with 95.2 ± 3.1% viability were obtained after isolation. At day 3, free hepatocytes displayed 99% viability, whereas microencapsulation in alginate and PEG-grafted alginate decreased viability to 62% and 48%, respectively. Albumin secretion and diazepam catabolism occurred in free and microencapsulated hepatocytes. Coencapsulation of hepatocytes with MSC significantly improved viability and albumin secretion at days 4 and 8 (*p* < 0.05). Coculture with MSC significantly increased and prolonged albumin secretion. In conclusion, we established a protocol for isolation and microencapsulation of high numbers of viable pig hepatocytes and demonstrated that the presence of MSC is beneficial for the viability and function of porcine hepatocytes.

## 1. Introduction

Hepatocyte cell transplantation is a potential solution to temporarily improve acute liver failure in patients that are waiting for a liver transplant [1]. Nevertheless, for patients with acute liver failure, the availability of suitable human liver cells remains a major problem. Transplantation of xenogeneic cells could be an adequate cell replacement treatment [2]. To circumvent the immune reaction toward the xenogeneic cells, encapsulation of cells in a semipermeable biocompatible polymer has been developed [3]. Micropores permit exchanges of nutrients, oxygen, and small-sized molecules. The promising first transplants with encapsulated hepatocytes in pig-to-rodent animal models showed that such transplanted encapsulated hepatocytes were still functional over several weeks [4–6]. In a pig-to-baboon model, 75% of baboons after induction of fulminant liver failure and subsequent transplantation with encapsulated hepatocytes recovered from liver injury [7], demonstrating further the therapeutic potential of such a treatment.

Previously, our laboratory established the first protocol for the isolation of primary pig and human hepatocytes and their encapsulation with alginate- (Alg-) poly-L-lysine (PLL) polymers [5, 6]. However, hepatocytes when encapsulated showed reduced functionality suggesting that polymers and microenvironmental conditions needed further optimization. Indeed, neonatal pig hepatocytes showed increased viability and functionality when previously reaggregated [4] or when heparin or hepatocyte growth factor was added during the encapsulation procedure [8]. The addition of PLL and collagen during encapsulation in alginate microcapsules prolonged viability and functionality, as demonstrated for cells of the human hepatocellular carcinoma cell line HepG2/C3A [9]. Further, coculture or coencapsulation with other cell types such as endothelial progenitor cells or fibroblasts showed enhanced viability and function of rat hepatocytes [10, 11].

Multipotent mesenchymal stromal cells, also called mesenchymal stem cells (MSC), support the growth of rodent hepatocytes by favoring cellular adhesion *in vitro* [12, 13] and *in vivo* [14]. Several studies in rodents have shown that cotransplantation of free primary hepatocytes with rat MSC prolonged graft function with a decrease in alanine transaminase (ALT), aspartate transaminase (AST), and bilirubin [15–17]. Further, coculture of rodent hepatocytes with MSC improved albumin secretion and metabolic capacities of hepatocytes [15, 16, 18].

The aim of this study was to optimize hepatocyte isolation to reach high yields of viable porcine hepatocytes and to assess viability, albumin secretory capacities, and metabolic functions after *in vitro* culture and microencapsulation. Furthermore, we aimed to assess the effect of human MSC on free and coencapsulated porcine hepatocytes, with particular attention to the effect on viability, albumin secretion, and cell morphology of pig hepatocytes.

## 2. Materials and Methods

### 2.1. Animals and Liver Harvesting

Ten-kilogram pigs were purchased from a local pig farm (M. Stirnimann, Apples, Switzerland) and were shortly housed in the animal facility of the University of Geneva, in compliance with all cantonal dispositions. Animal research was performed following protocols approved by the Geneva cantonal veterinary authorities (license GE/79/15). Pigs were fasted overnight and premedicated with azaperone (1 mg/kg) and atropine (0.05 mg/kg) + dormicum (0.5 mg/kg) by intramuscular injection, prior to anesthesia with isoflurane, fentanyl (0.1 mg), and atracurium (1 mg/kg). After heparin injection (5000 U/L) and disinfection, an abdominal incision was performed, followed by gallbladder removal and perfusion with cold preservation medium IGL-1 (3.5 L) (Institut Georges Lopez, Lissieu, France) through the portal vein and the hepatic artery. A total hepatectomy was performed, and the liver was stored on ice for a maximum of 30 min prior to hepatocyte isolation.

### 2.2. Cell Isolation and Culture

**Figure 1:**
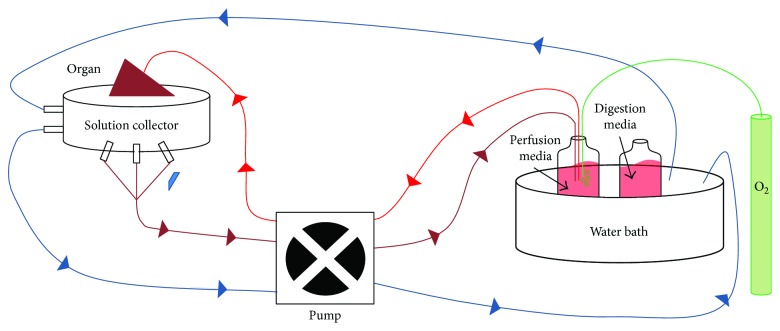
Liver perfusion and digestion system. The isolated liver is maintained at 37°C by water circulation (in blue) supplied by a water bath kept at 37°C. Perfusion and digestion media are maintained at 37°C and oxygenated through the oxygen cylinder (in green). Thereafter, infusion of perfusion media followed by digestion media (in red) into the liver is performed via the vena cava. Flow-through of both media (in brown) is recovered and discarded.

Isolation of porcine hepatocytes was performed as previously described [5]. Briefly, the liver was perfused through the vena cava with liver perfusion medium for 15 minutes at 37°C (Life Technologies, Carlsbad, CA, USA); thereafter, digestion media containing collagenase NB 4 Standard Grade (3 g/L; Serva, Heidelberg, Germany) were infused for 25 minutes through the vena cava (Figure 1). Mechanical destruction and filtration of the liver through a 100 *μ*m stainless steel mesh were performed to obtain a hepatocyte suspension, as previously described [5]. The hepatocyte cell suspension was washed twice using hepatocyte wash medium (Life Technologies) and centrifuged at 68*g* for 10 minutes; cells were counted and then cultured in DMEM/F12 (Life Technologies), supplemented with dexamethasone (0.4 *μ*g/mL, Sigma-Aldrich, Buchs, Switzerland), insulin (0.02 E/U/mL, Novo Nordisk, Plainsboro, NJ, USA), apo-transferrin (5 *μ*g/mL, Sigma), penicillin (100 IU/mL) and streptomycin (100 mg/mL) (P-S; Gibco-Thermo Fisher, Waltham, MA, USA), and 10% of autologous serum, obtained after clotting and high-speed centrifugation of porcine blood.

MSC were isolated from the femoral head of the patients undergoing total hip replacement. MSC were characterized by surface receptor expression using FACS analysis and by their capacity to differentiate into osteoblasts, chondrocytes, and adipocytes as shown previously [19, 20]. All patients gave informed consent, and the experimental procedure was approved by the local ethical committee of the University Hospitals of Geneva (NAC 01-015). Briefly, after isolation, through mechanical destruction and Ficoll density gradient centrifugation, MSC were cultured in Iscove's modified Dulbecco's medium (Gibco-Thermo Fisher) with 10% fetal calf serum (Gibco-Thermo Fisher), P-S, and 10 ng/mL platelet-derived growth factor BB (PDGF-BB; PeproTech EC Ltd., London, UK). MSC were used from passages 2 to 5.

### 2.3. Polymer Synthesis

Sodium-Alg Kelton High Viscosity (lot number 61650A; *η* = 813 mL·g^−1^ in 0.1 M NaCl, *T* = 25°C, and G/M = 0.6) was obtained from Kelco (San Diego, CA, USA). Commercial reagents (Fluka, Sigma, Switzerland; TCI Europe, Zwijndrecht, Belgium) were used without further purification. Unless mentioned otherwise, all reactions were performed under argon atmosphere (1 atm). Anhydrous solvents were obtained by filtration (PureSolv MD 5, Innovative Technology, Oldham, UK). Glassware was dried for 12 h in an oven (*T* > 100°C) or under vacuum with a heat gun (*T* > 200°C). Nuclear magnetic resonance (NMR) spectra were recorded on Bruker Avance III-400, Bruker Avance-400, or Bruker DRX-400 spectrometers at room temperature (rt) (400 MHz) (Bruker, Billerica, MA, USA). ^1^H frequency is at 400.13 MHz. Chemical shifts are expressed in parts per million (ppm) and coupling constants (*J*) in hertz (Hz). The solvent used for NMR spectroscopy was deuterated water.

Polymer Alg-poly(ethylene-glycol)- (PEG-) SH (Alg-PEG-SH) was prepared according to a procedure that we recently established [21]. A solution of thiobarbituric acid- (TBA-) Alg [22] (200 mg, 0.478 mmol) in dimethyl sulfoxide (DMSO) (40 mL) was stirred for 12 hours at 22°C. To this solution, 1,1′-carbonyldiimidazole (CDI) (77.4 mg, 0.478 mmol) was added, and the mixture was stirred at 22°C for 0.5 h. Acetone (80 mL) was added, and the resulting precipitate was filtered and washed with acetone (20 mL, 3 times). The solid was dried for 15 minutes under vacuum at 40°C and dissolved in distilled water (20 mL). A solution of *α*-amino-*ω*-azido PEG (H_2_N-PEG-SH) (98.8 mg, 95.8 *μ*mol) in distilled water (1 mL) was added, and the solution was stirred for 2 hours at 22°C. NaOH (0.05 M aqueous solution) was added until reaching pH 11.0, and the solution was transferred to a dialysis membrane and dialyzed against water. After one water change, tris(2-carboxyethyl)phosphine (TCEP) (0.1 M, 1 mL) was added in the dialysis tube, and the dialysis was continued against water for 3 days. An aqueous solution of NaHCO_3_ was added until reaching pH 7. The solution was filtered (70 *μ*m and 0.22 *μ*m) and freeze-dried to afford Alg-PEG-SH as a white solid (106.7 mg) (Supplementary Figure 1). The percentage of grafting, determined by H-NMR, was 30.3%. The viscosity of the polymer (measured in distilled water at 22°C) was 204.6 mPa·s.

### 2.4. Hepatocyte Microencapsulation

Hepatocytes and MSC were coencapsulated in a ratio 1 : 1, keeping in mind that hepatocytes are in contact with nonparenchymal cells in liver plates [23]. Hepatocytes (3 × 10^6^/mL of polymer) with or without MSC (3 × 10^6^/mL of polymer) were gently mixed with calcium-alginate (Ca-Alg), under sterile conditions. Alg-PEG-SH (3% diluted in 3-(N-morpholino)propanesulfonic acid (MOPS)) was also used to encapsulate hepatocytes. All polymers were prepared by collaborators of the École Polytechnique Fédérale de Lausanne. Microbeads were produced using the Buchi Encapsulator B-395 Pro (Büchi Labortechnik AG, Flawil, Switzerland). Ionic and covalent cross-linking occurred immediately after bead immersion into the gelation bath, composed of 10 mM MOPS with pH = 7.4 and 100 mM CaCl_2_. After formation, microspheres were straightaway collected by filtration to remove the gelation bath, washed with 0.9% NaCl, and immediately cultured in complete DMEM/F12 medium. The diameter of resulting beads was 500–600 *μ*m for Ca-Alg and 400–500 *μ*m for Alg-PEG-SH [21].

### 2.5. Viability of Primary and Encapsulated Cells

Viability and cell death of free and microencapsulated cells (0.2 × 10^6^ cells/1 mL/24-well plate) were analyzed using fluorescein diacetate (FDA) and propidium iodide (PI) for the staining of viable and dead cells, respectively, as previously described [24]. Images were acquired using a fluorescent microscope and LAS V4.5 software (Leica Microsystem, Heerbrugg, Switzerland). Quantification of cell viability was performed 3 days after culture using ImageJ (https://imagej.nih.gov/ij) and expressed as a percentage where the sum of the value measured, for FDA-positive and PI-positive cells in the field of view, was set as 100%.

### 2.6. Measurement of Albumin Secretion

Hepatocytes (0.2 × 10^6^ cells) alone or with MSC (0.2 × 10^6^ cells, ratio 1 : 1) and microencapsulated or not were seeded with 1 mL complete DMEM/F12 medium in a 24-well Corning Primaria Cell Culture Multiwell Plate (Fisher Scientific, Hampton, NH, USA). Prior to albumin secretion assays, cells were washed and serum-starved overnight to remove pig albumin. Every 24 hours, medium was collected from day 2 to day 8; samples were frozen until albumin measurements. Albumin was measured following the manufacturer's instructions using an albumin pig ELISA kit (Abcam, Cambridge, UK).

### 2.7. Measurements of Drug Metabolism

1 *μ*g/mL of diazepam was added to free and encapsulated hepatocytes (0.2 × 10^6^ cells/1 mL/24-well plate) for 6 hours at days 1, 3, and 7. Supernatants were collected and frozen until metabolite measurements. Prior to the quantitative analysis, protein precipitation was performed on the samples using a solution of methanol : ethanol at a ratio 1 : 1. Then, samples were centrifuged at 14,000*g* for 15 minutes. Samples were lyophilized with a SpeedVac system and reconstituted in 10% methanol. Quantitative analysis was performed by LC-MS/MS with a selected reaction monitoring (SRM) mode. The UltiMate 3000 LC system from Dionex coupled to a triple quadripole 5500 QTRAP system from AB Sciex was used. The LC separation was conducted on a Kinetex C18 column (50 × 2.1 mm (i.d.)) (Phenomenex). The mobile phases were made of A (H_2_O with 0.1% formic acid) and B (ACN + 0.1% formic acid). The flow rate was 0.6 mL/min. The SRM transitions used for the quantification of the diazepam and its respective metabolites are referred in Supplementary Table 1.

### 2.8. Immunofluorescence Staining on Cultured Cells

Hepatocytes (0.2 × 10^6^ cells) alone or with MSC (0.2 × 10^6^ cells, ratio 1 : 1) were seeded on 12 mm coverslips in a 24-well plate in 1 mL of complete F12 medium. After 3 days, cells were washed with phosphate-buffered saline (PBS) and fixed with a 10% formalin solution (Sigma-Aldrich, Buchs, Switzerland) for 12 min. Cells were then permeabilized with Triton X-100 0.1% diluted in PBS for 15 min, and epitopes were blocked using 0.5% bovine serum albumin (BSA) for 30 min. Hepatocytes were stained with an anti-pig albumin antibody (Abcam) diluted to 1/200 and a secondary Alexa Fluor 555 goat anti-rabbit antibody (Life Technologies) diluted to 1/500. MSC were stained with a mouse anti-human vimentin antibody diluted to 1 : 50 (Dako, Glostrup, Denmark) and a secondary Alexa Fluor 488 goat anti-mouse antibody (Life Technologies). For 5-ethynyl-2′-deoxyuridine (Edu) staining, the Click-iT EdU Alexa Fluor 488 Imaging Kit (Thermo Fisher) was used following the manufacturer's instructions. Coverslips were mounted using VECTASHIELD mounting medium with 4′,6-diamidino-2-phenylindole (DAPI) (Vector Laboratories, Cambridgeshire, UK). Images were acquired using a fluorescence microscope and LAS V4.5 software (Leica Microsystems).

### 2.9. Measurements of Cytokines in Cell Culture Supernatants

MSC alone or with hepatocytes (0.2 × 10^6^ cells/1 mL/24-well plate) from 2 different donors were cultured in complete hepatocyte medium for 2 days. Cells were washed and cultured for 24 h in medium without FCS. Cell culture supernatants were collected and frozen until cytokine measurements. Cytokines were screened using Proteome Profiler, Human Cytokine Array (R&D Systems, Bio-techne, Minneapolis, MN, USA), following the manufacturer's procedures. Quantification of cytokine measurements was performed using ImageJ (https://imagej.nih.gov/ij) and expressed as mean pixel density.

### 2.10. Statistical Analysis

Results are expressed as mean ± standard error of the mean (SEM). Numbers of experiments are indicated in the legend of each figure. GraphPad Prism software was used. Values of hepatocytes alone or cocultured with MSC were compared using the ratio paired *t*-test, and differences were considered significant when *p* < 0.05(∗), *p* < 0.01(∗∗), and *p* < 0.001(∗∗∗).

## 3. Results

### 3.1. Hepatocyte Isolation and Cell Yield and Viability

**Figure 2:**
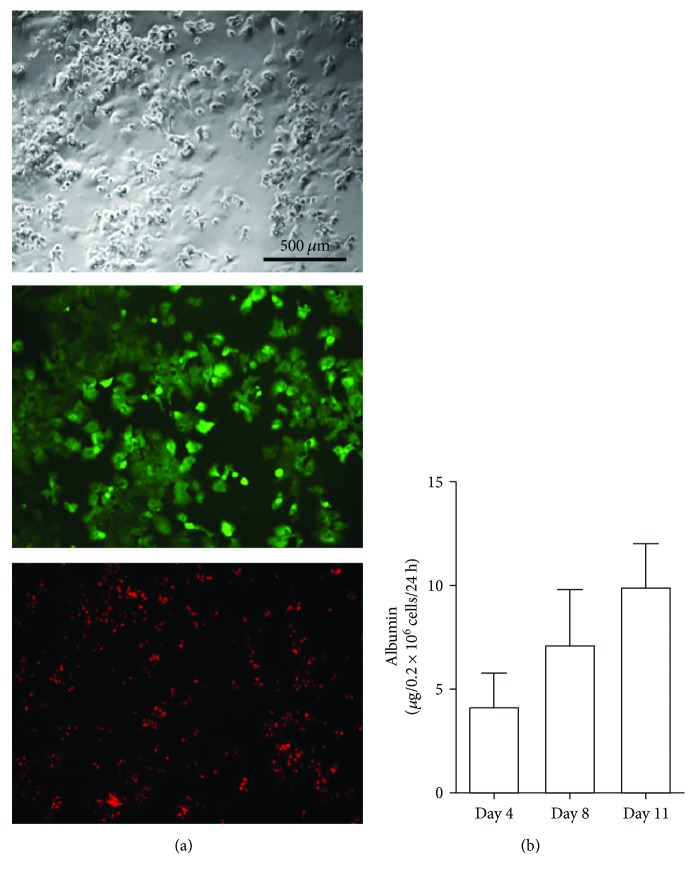
Hepatocyte viability and function after freezing and thawing. (a) The upper panel presents the bright-field image of thawed hepatocytes in culture after 3 days, the middle panel presents viable cells (FDA staining), and the lower panel presents nonviable cells (PI staining). (b) Albumin was measured by ELISA in the supernatant of cultured hepatocytes at days 4, 8, and 11. The experiment is performed in duplicates.

After surgical recovery of the livers from 10-kilogram pigs (*n* = 12), 9.2 ± 3.6 × 10^9^ total hepatocytes were isolated with a yield of 27.9 ± 9.9 × 10^6^ cells/g (Table 1). Immediately after hepatocyte isolation and purification, viability was of 95.2 ± 3.1%, showing that the harvesting protocol allowed the isolation of high yields of viable porcine hepatocytes from 10-kilogram pigs. For the ultimate purpose to use frozen-stored porcine hepatocytes for transplantation, several batches of hepatocytes (10 × 10^6^ hepatocytes in 1 mL of cryopreservation media) were frozen in 10% DMSO and ready-to-use CryoStor CS10 (Sigma-Aldrich) cryopreservation media. CryoStor-frozen cells were thawed in complete medium. After cell centrifugation at 68*g*, pellets of cells were resuspended in complete media. Cells were stained with trypan blue, and viability was evaluated under bright-field microscopy. 0.9 × 10^6^ hepatocytes were obtained, corresponding to 90% of total cells. After seeding and culture, hepatocytes maintained viability at 3 days (Figure 2(a)); however, no viable hepatocytes were recovered when frozen with DMSO-containing medium. Furthermore, thawed hepatocytes after freezing in CryoStor maintained albumin secretion at days 4, 8, and 11 (Figure 2(b)).

### 3.2. Hepatocyte Viability Is Maintained after Ca-Alg and Alg-PEG-SH Microencapsulation

**Figure 3:**
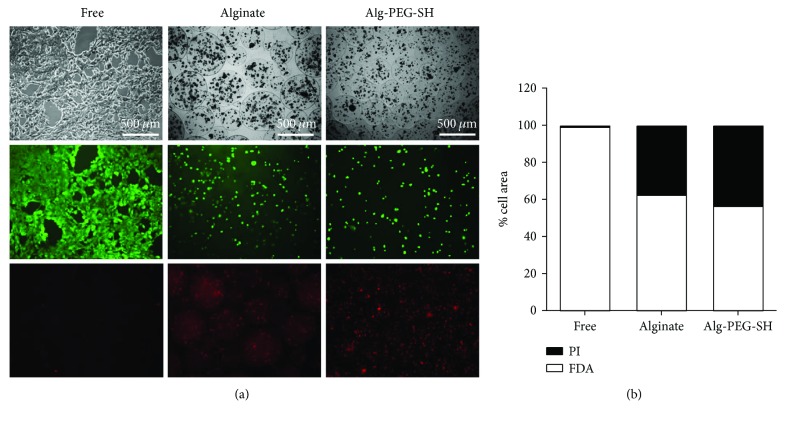
Viability of free and microencapsulated hepatocytes. (a) Representative images at day 3 for free, Alg-microencapsulated, and Alg-PEG-SH-microencapsulated hepatocytes. Upper panels present bright-field images, middle panels present viable cells (FDA staining), and lower panels present nonviable cells (PI staining). (b) Quantification of viable cells (FDA) and nonviable cells (PI) at day 3. Values are expressed as % of the total cell area (*n* = 6). Quantification has been performed using ImageJ.

During the *in vitro* culture of free and encapsulated hepatocytes, we assessed hepatocyte viability and mortality, using FDA-PI staining each day. *In vitro*, free hepatocytes maintained viability up to 10 days in adherent culture conditions; Alg- and Alg-PEG-SH-microencapsulated hepatocytes remained viable up to 7 days. After 3 days of culture, free hepatocytes were 99% viable with minimal cell death (1%; Figures 3(a) and 3(b)). After microencapsulation in beads of 400–600 *μ*m diameters, Alg-microencapsulated hepatocytes maintained 62% viability and Alg-PEG-SH-encapsulated hepatocytes maintained 56% viability (Figures 3(a) and 3(b)). These results demonstrate that microencapsulation of porcine hepatocytes with both types of polymers allows survival of up to 50% of the microencapsulated pig hepatocytes.

### 3.3. Albumin Secretion Is Maintained in Hepatocytes after Microencapsulation

**Figure 4:**
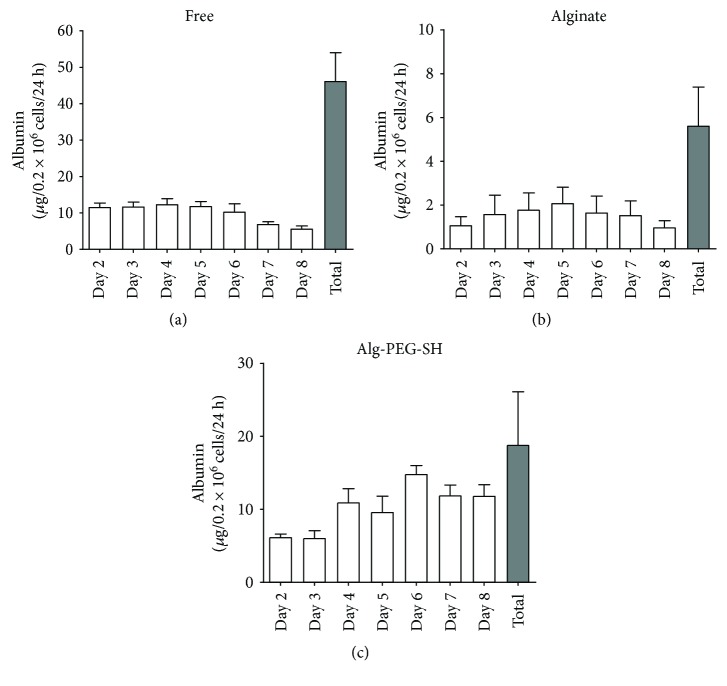
Albumin secretion from free and microencapsulated hepatocytes. Albumin was measured by ELISA in the supernatant of free (*n* = 9) (a), alginate-encapsulated (*n* = 3) (b), and Alg-PEG-SH-encapsulated (*n* = 3) (c) hepatocytes. White bars represent albumin secretion during 24 h from day 2 to day 8, and grey bars represent the total albumin secreted during 8 days.

To assess hepatocyte functionality after microencapsulation, albumin secretion was measured starting from day 2 to day 8 in free, Alg-microencapsulated, and Alg-PEG-SH-microencapsulated hepatocytes. Measurements at 24 hours showed that free hepatocytes secreted 10–12 *μ*g/mL/24 hours albumin until day 6, and at days 7 and 8, albumin secretion was halved (Figure 4(a)). The total amount of albumin secreted during 8 days was around 46 *μ*g/mL in free cultured hepatocytes (Figure 4(a), grey bar). The albumin secretion from free hepatocytes isolated from 9 livers was at similar levels. Alg-microencapsulated hepatocytes secreted lower amounts of albumin (2–1.5 *μ*g/mL/24 h), with a maximum amount measured at day 4 to day 6. Despite the decrease in the amount of albumin measured, secretion still persisted until day 8 (Figure 4(b)). Alg-PEG-SH-microencapsulated hepatocytes secreted albumin until day 8 in similar amounts when compared with free cultured hepatocytes (Figure 4(c)), with the highest secretion at day 6. These results show that microencapsulated hepatocytes secrete albumin, which is maintained up to 8 days in free, Alg-microencapsulated, and Alg-PEG-SH-microencapsulated hepatocytes.

### 3.4. Microencapsulated Hepatocytes Still Display the Capacity to Metabolize Diazepam

**Figure 5:**
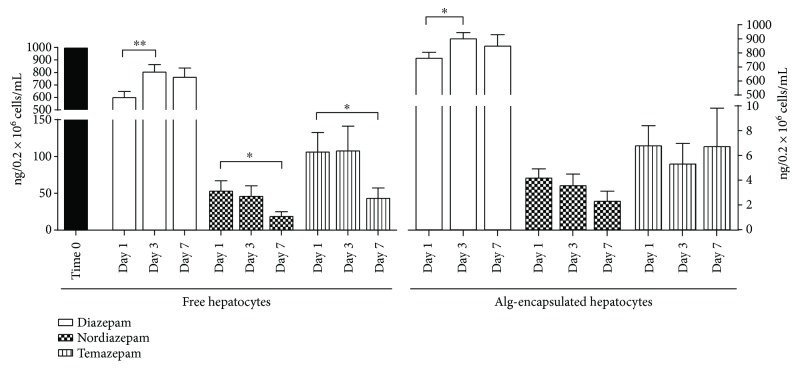
Diazepam metabolism in free and alginate-microencapsulated hepatocytes. Diazepam (white bars) was added at days 1, 3, and 7 on free and alginate-encapsulated hepatocytes, and supernatant was retrieved after 6 hours of culture. Diazepam and its metabolites nordiazepam and temazepam were measured by LC-MS/MS in 4 independent experiments. ^∗^ *p* < 0.05, ^∗∗^ *p* < 0.01.

To evaluate the metabolic capacities of free and microencapsulated hepatocytes, diazepam was added at days 1, 3, and 7 to hepatocytes for 6 hours and the metabolites nordiazepam and temazepam were measured. As shown in Figure 5, diazepam decreased similarly in both free and Alg-microencapsulated hepatocytes (white bars). At day 1, metabolic capacities were maximal; nordiazepam and temazepam were 49 ng/mL and 93.4 ng/mL, respectively, in free hepatocytes and 3.5 ng/mL and 5.6 ng/mL, respectively, in microencapsulated hepatocytes. The amounts of metabolites released decreased progressively between day 3 and 7, for both free and Alg-microencapsulated cells. This result suggests that, although lower amounts of diazepam metabolites were measured in the supernatant of encapsulated hepatocytes, hepatic metabolic function for diazepam still occurred in microencapsulated hepatocytes during the first week of *in vitro* culture.

### 3.5. Hepatocytes Show Increased Viability and Albumin Secretion When Coencapsulated with MSC

**Figure 6:**
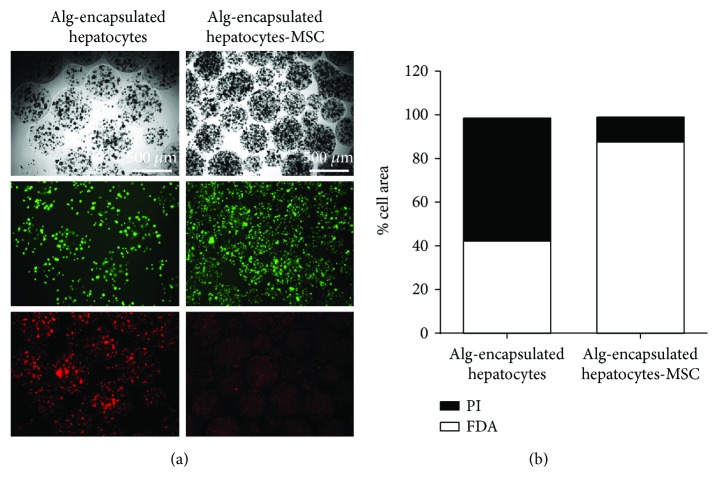
Viability of coencapsulated hepatocytes and MSC. (a) Representative images of Alg-encapsulated hepatocytes alone or with MSC after 4 days of culture. Upper panels present bright-field images, middle panels present viable cells (FDA staining), and lower panels present nonviable cells (PI staining). (b) Quantification at day 3. Values are expressed as % of the total cell area (*n* = 2). Quantification has been performed using ImageJ.

**Figure 7:**
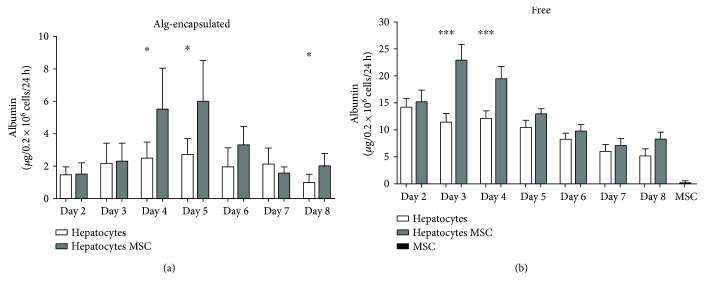
Albumin secretion of hepatocytes coencapsulated and cocultured with MSC. Albumin secretion was measured by ELISA in the supernatant of cell culture after 24 hours from day 2 to day 8. (a) Alg-microencapsulated hepatocytes alone (white bars) or with MSC (grey bars), measured in 2 independent experiments. (b) Free hepatocytes alone (white bars) or with MSC (grey bars), measured in 5 independent experiments. ^∗^ *p* < 0.05, ^∗∗∗^ *p* < 0.001.

Trophic molecules secreted by MSC have been described to be beneficial for cell function in acute liver failure [25]. To assess whether MSC could provide a beneficial effect on microencapsulated hepatocytes, both were coencapsulated in an Alg microsphere. To assess viability, FDA-PI staining was performed at day 3. We observed that in capsules containing hepatocytes and MSC, total cell death was decreased considering the total number of PI- and FDA-stained cells. Hepatocyte survival reached 90% whereas hepatocytes alone reached 40% (Figures 6(a) and 6(b)). Further, to assess the functionality of hepatocytes coencapsulated with MSC, albumin secretion was measured each day starting at day 2 until day 8. In the presence of MSC (grey bars), albumin secretion was significantly higher at days 4, 5, and 8 (*p* < 0.05, Figure 7(a)) for hepatocytes coencapsulated with MSC than for microencapsulated hepatocytes alone (white bars). To assess the importance of cellular contact between hepatocytes and MSC, coculture was performed and albumin secretion was measured in the supernatants. At days 3 and 4, albumin secretion was significantly higher in hepatocytes with the presence of MSC (grey bars, *p* < 0.001) compared to hepatocytes alone (Figure 7(b), white bars). Noteworthy, MSC alone secreted only limited amounts of albumin (grey bars). Further, the presence of MSC allowed prolonging albumin secretion up to day 15 (Supplementary Figure 2). Diazepam metabolism of hepatocytes cocultured with MSC was similar to that of hepatocytes cultured alone (Supplementary Figure 3), suggesting that the cell-cell contact between hepatocytes and MSC does not affect metabolic function of hepatocytes at short time. All together, these results show that coencapsulation of hepatocytes with MSC improves and prolongs significantly albumin secretion from porcine hepatocytes.

### 3.6. Human MSC and Pig Hepatocyte Distribution in Coculture

**Figure 8:**
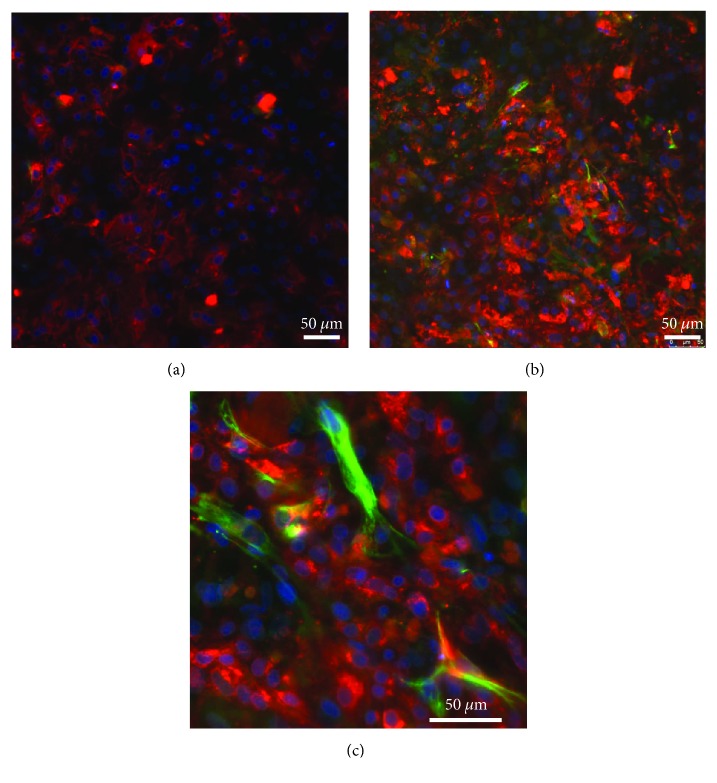
Distribution of porcine hepatocytes and human MSC in coculture. Cells were cultured for 2 days and then serum-starved to avoid nonspecific staining. Immunofluorescence was performed on cells after 3 days of culture. (a) Hepatocytes are stained for porcine albumin (in red). (b) Coculture of hepatocytes and MSC. MSC are stained for human vimentin (in green). (c) Coculture of hepatocytes and MSC in a higher magnification (×40). Images are representative of 2 independent pig hepatocyte isolations.

Coculture of hepatocytes and MSC was used to analyze the cell distribution after 3 days of culture. MSC were stained for the cytoskeleton protein vimentin (in green) and hepatocytes for albumin (in red) (Figure 8(a)). Hepatocytes alone formed a typical adherent epithelial cell layer (Figure 8(a)). In coculture with MSC, hepatocytes appeared in small cell clusters (hepatocyte doublets or triplets), with the presence of MSC intermingled throughout the hepatocyte culture (Figures 8(b) and 8(c)).

### 3.7. Cytokine Screening in Supernatants of Human MSC Cultured Alone or with Porcine Hepatocytes

**Figure 9:**
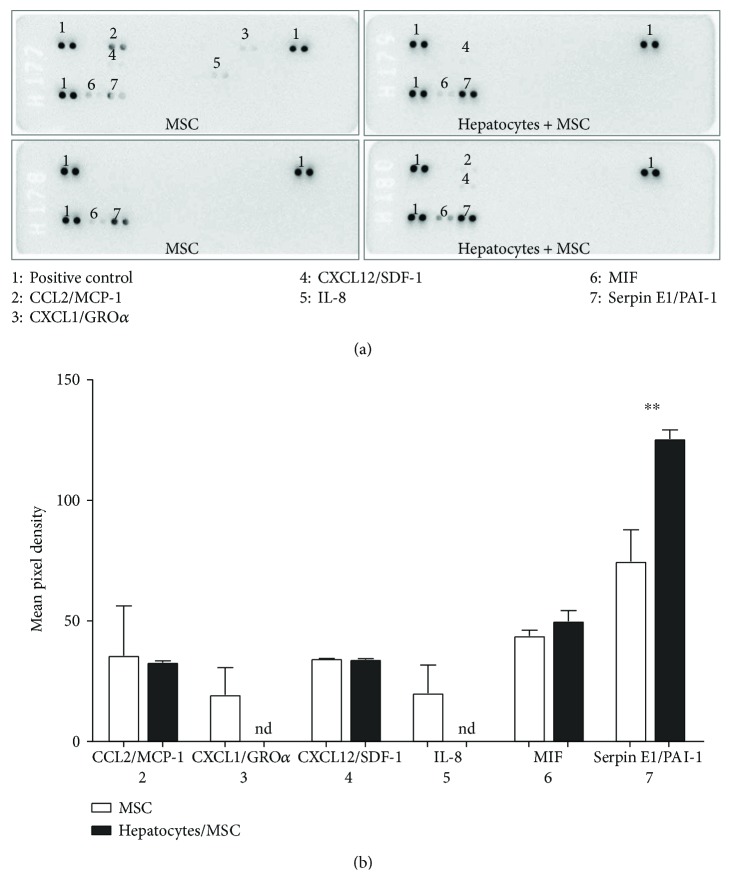
Profiling of human cytokines using cell culture supernatants of MSC alone and MSC cocultured with hepatocytes. (a) Human cytokines were analyzed using antibody membranes in MSC alone and MSC cocultured with hepatocytes (0.2 × 10^6^ cells/mL/24-well plate) obtained from 2 different donors. (b) Spots were quantified using ImageJ and expressed as mean pixel density. Cytokines secreted from MSC cultured alone (white bars) and cytokines secreted from MSC cocultured with hepatocytes (black bars). ^∗∗^ *p* < 0.01.

Cytokines secreted from MSC alone or cocultured with hepatocytes were screened using a human antibody array to evaluate an eventual cytokine response from MSC induced by hepatocytes. Several cytokines were detected in the 24 h supernatants (Figure 9(a)). CCL2, CXCL12, and macrophage migration inhibitory factor (MIF) were present in both cell culture supernatants. CXCL1 and IL-8 were only detectable in the supernatant of MSC from 1 of 2 donors. Interestingly, serpin E1 was present in the supernatants of MSC cultured alone, but its presence was significantly increased in MSC cocultured with hepatocytes (Figure 9(b)). These results show that MSC secrete only few inflammatory cytokines and this also occurs during coculture with porcine hepatocytes suggesting further that MSC do not increase the expression of proinflammatory cytokines when in contact with porcine hepatocytes at a short term.

## 4. Discussion

Acute liver failure has high mortality rate. Liver transplantation, which has to occur in the few days following liver destruction, remains the only treatment with lifelong immunosuppression thereafter [1]. Additionally, the availability of an adequate human liver is not guaranteed, demonstrating the need for new therapeutic options. Porcine hepatocyte cell transplantation might present an alternative solution to overcome acute liver failure damages by replacing the metabolic function of the liver until its recovery. Earlier studies in mice with acetaminophen- and hepatectomy-induced fulminant liver failure showed that transplantation of microencapsulated human or porcine hepatocytes increased survival rates of mice [5, 6] and the first protocols for porcine hepatocyte isolation and encapsulation with Alg-PLL capsules were applied [5, 6].

Here, we developed an optimized high-yield porcine hepatocyte isolation protocol from 10-kilogram pigs. Hepatocytes were encapsulated in recently developed biomaterials which showed an improved biocompatibility, compared to previous polymers. Currently, new biocompatible biomaterials which allow the production of long-term stable microspheres for hepatocyte encapsulation are under investigation. Long-term stability remains an important issue to prevent immunoreaction due to microcapsule disaggregation. Several types of polymers are under investigation. Durkut and collaborators showed that rat hepatocytes maintained comparable viability in free and encapsulated conditions in Alg-chitosan-Alg microcapsules [26]. However, this study did not focus on microbead stability. Furthermore, another study reports that hepatocytes isolated from rats and encapsulated in PEG did not survive after encapsulation [11]. Therefore, improvements are needed to maintain viability of primary hepatocytes which are highly sensitive to environmental conditions. Our data shows that using Alg and a hybrid Alg-PEG-SH biomaterial for encapsulation allows maintaining 56% viability of encapsulated hepatocytes, compared to hepatocytes alone. The diminished hepatocyte viability was reflected by decreased albumin secretion and metabolic activity of diazepam, but the function was partially maintained after microencapsulation when compared to that of free cultured hepatocytes. Actually, the initial amount of diazepam decreased in free and encapsulated conditions; however, the quantity of metabolites did not significantly increase in the supernatants of encapsulated hepatocytes. An issue to be considered is that molecule diffusion might be delayed; indeed, albumin and diazepam and its metabolites may remain longer inside the microsphere, making it difficult to compare the amounts of molecules released after 6 hours from free and encapsulated hepatocytes; however, cell viability and molecule dispersion were maintained.

A number of options to improve hepatocyte viability and function have been proposed. The supplementation with modulators like heparin or collagen during microencapsulation improves albumin and urea synthesis and increases hepatocyte viability [8, 9]. For rat hepatocytes, coencapsulation with other cells such as endothelial progenitor cells improves albumin and urea secretion [10]. MSC express low levels of human leukocyte antigen classes 1 and 2 and costimulatory molecules on their surface, conferring MSC low immunogenicity [27]. Furthermore, MSC have the capacity to suppress cell activation and proliferation of immune cells, in particular T cells, B cells, and dendritic cells [28]. The immune response of MSC to xenogeneic hepatocytes was analyzed using an antibody array for screening human cytokines. Under our culture conditions, with two different donor-derived MSC populations, we detected mainly 2 types of cytokines, CCL2 and MIF. CCL-2 and MIF are implicated in the chemotactic activity of immune cells and cell-mediated immunity, respectively [29, 30]. Furthermore, serpin E1 is increasingly detected in coculture conditions with hepatocytes. Serpin E1 is a serine protease inhibitor [31] secreted by MSC as demonstrated by Daltro et al., where serpin E1 secreted by MSC was involved in recovery of cardiac disturbances [32]. We did not observe major changes in the cytokine profile of MSC which further suggests that MSC, when exposed to xenogeneic hepatocytes, do not display a higher immunogenicity.

Moreover, MSC have antifibrotic properties [20] and have been successfully used to treat liver failure [33]. Therefore, we explored the effect of human MSC on porcine hepatocyte survival and function. The present results show that MSC significantly improve hepatocyte viability and albumin secretion in coculture and coencapsulated conditions. However, others have reported that for 3D cultures of human MSC and human hepatocytes, there was no effect on albumin secretion, but rather hepatocyte was compacted in morphology and there was a phenotypic stability [34]. Also, human MSC potentiated hepatotrophic and antiapoptotic genes in human primary hepatocytes [35], with the accumulation of hepatocytes in the G2/S phase of the cell cycle, meaning that they are prone to proliferation [18]. In line with these results, in the present study, hepatocytes in coculture with MSC tend to show extended survival compared to microencapsulated hepatocyte alone.

The mechanism how MSC exert their beneficial effects needs further investigations and might not solely be due to paracrine effects. Interestingly, the systemic injection of extracellular vesicles, derived from bone marrow MSC after *in vitro* culture, reduced hepatic injury and improved mice survival [25], suggesting that such vesicles contain molecules either acting directly on liver cells or modulating the immune system. Moreover, the evaluation of the secretome of human MSC evidenced a correlation between vascular endothelial growth factor and cell proliferation, development processes, and immune processes. Also, systemic injection of conditioned medium of MSC in liver-injured mice improved survival [36]. In our experimental conditions, we found increased albumin secretion when hepatocyte and MSC were cultured together; this might not be due exclusively to cellular interactions but might also include the effect of secreted molecules. In particular, in encapsulated condition, hepatocytes and MSC are distributed throughout the microcapsules (Figure 6(a)); both improved microenvironment and paracrine signaling through secreted molecules might contribute to the increased viability and functionality of hepatocytes.

## 5. Conclusion

In conclusion, we performed high-yield porcine hepatocyte isolations that allow obtaining high quantities of viable hepatocytes. Further, we used a newly developed polymeric biomaterial which allowed the maintenance of hepatocyte viability, albumin secretion, and drug metabolic functions up to 8 days. Furthermore, hepatocyte coencapsulation with MSC increased and prolonged hepatocyte viability, suggesting that cell-to-cell contact and paracrine effects are beneficial for hepatocyte function and survival. This optimized protocol for porcine hepatocyte isolation and microencapsulation can now be used for further experimental cell transplantation research in liver diseases to evaluate further the potential of encapsulated hepatocytes for the treatment of acute liver failure in humans.

## Figures and Tables

**Table 1:** Hepatocyte isolation.

Total number of hepatocytes	9.2 ± 3.6 × 10^9^
Cells/g	27.9 ± 9.9 × 10^6^
Viability	95.2 ± 3.1

## Abbreviations

Alg: Alginate
Alg-PEG-SH: Alginate-poly(ethylene-glycol)-SH
ALT: Alanine transaminase
AST: Aspartate transaminase
Ca-Alg: Calcium-alginate
DMSO: Dimethyl sulfoxide
Edu: 5-Ethynyl-2′-deoxyuridine
FDA: Fluorescein diacetate
MIF: Macrophage migration inhibitory factor
MSC: Multipotent mesenchymal stromal cells
NMR: Nuclear magnetic resonance
PBS: Phosphate-buffered saline
PEG: Poly(ethylene-glycol)
PI: Propidium iodide
PLL: Poly-L-lysine
SEM: Standard error of the mean
SRM: Selected reaction monitoring.

## Supplementary Materials

Supplementary 1 Supplementary Figure 1: polymer synthesis.

Supplementary 2 Supplementary Table 1: mean (±SD) yield and viability of hepatocytes isolated from 12 pigs.

Supplementary 3 Supplementary Figure 2: albumin secretion of hepatocytes cocultured with MSC until day 15. Albumin secretion was measured by ELISA in the supernatant of cell culture after 24 hours from day 2 to day 15. Free hepatocytes alone (white bars) or with MSC (grey bars), measured in 2 independent experiments.

Supplementary 4 Supplementary Figure 3: diazepam metabolism in hepatocytes cocultured with MSC. Diazepam was added at days 1, 3, and 7 and supernatant was retrieved after 6 hours of culture. Diazepam (white bars) and their metabolites nordiazepam, oxazepam, and temazepam were measured by LC-MS/MS in free hepatocytes and hepatocytes cocultured with MSC 4 independent experiments.
