# Supplementary material for: Beneficial Effects of Human Mesenchymal Stromal Cells on Porcine Hepatocyte Viability and Albumin Secretion
Source: J Immunol Res. 2018 Jan 11;2018:1078547. doi: 10.1155/2018/1078547 (PMC5822000; doi:10.1155/2018/1078547)
Supplement: Supplementary 2 — Supplementary Table 1: mean (±SD) yield and viability of hepatocytes isolated from 12 pigs. [file 1078547.f2.docx]

**Supplementary table 1**

| Analytes name | Q1 (*m/z*) | Q3 (*m/z*) | Collision energy (CE) |
| --- | --- | --- | --- |
| Diazepam | 285.0 | 241.0 | 55 |
| Nodiazepam | 271.0 | 140.0 | 55 |
| Oxazepam | 287.0 | 241.0 | 55 |
| Temazepam | 301.0 | 255.0 | 55 |
